# Cytogenetics in Fanconi Anemia: The Importance of Follow-Up and the Search for New Biomarkers of Genomic Instability

**DOI:** 10.3390/ijms232214119

**Published:** 2022-11-15

**Authors:** Lismeri Wuicik Merfort, Mateus de Oliveira Lisboa, Luciane Regina Cavalli, Carmem Maria Sales Bonfim

**Affiliations:** 1 Cytogenetics Laboratory, Universidade Federal do Paraná (UFPR), Curitiba 80060-240, Brazil; 2 Core for Cell Technology, Pontifícia Universidade Católica do Paraná (PUCPR), Curitiba 80215-901, Brazil; 3 Research Institute Pelé Pequeno Príncipe, Faculdades Pequeno Príncipe (FPP), Curitiba 80230-020, Brazil

**Keywords:** Fanconi Anemia, cytogenetics, chromosomes, genomic instability, bone marrow, cancer

## Abstract

Fanconi Anemia (FA) is a disease characterized by genomic instability, increased sensitivity to DNA cross-linking agents, and the presence of clonal chromosomal abnormalities. This genomic instability can compromise the bone marrow (BM) and confer a high cancer risk to the patients, particularly in the development of Myelodysplastic Syndrome (MDS) and Acute Myeloid Leukemia (AML). The diagnosis of FA patients is complex and cannot be based only on clinical features at presentation. The gold standard diagnostic assay for these patients is cytogenetic analysis, revealing chromosomal breaks induced by DNA cross-linking agents. Clonal chromosome abnormalities, such as the ones involving chromosomes 1q, 3q, and 7, are also common features in FA patients and are associated with progressive BM failure and/or a pre-leukemia condition. In this review, we discuss the cytogenetic methods and their application in diagnosis, stratification of the patients into distinct prognostic groups, and the clinical follow-up of FA patients. These methods have been invaluable for the understanding of FA pathogenesis and identifying novel disease biomarkers. Additional evidence is required to determine the association of these biomarkers with prognosis and cancer risk, and their potential as druggable targets for FA therapy.

## 1. Introduction

Fanconi Anemia (FA) was first described in 1927 by Dr. Guido Fanconi, a Swiss pediatrician, after he observed a family with three brothers with physical congenital disabilities who died from a condition resembling pernicious anemia, i.e., Macrocytic red cells and pancytopenia, along with several physical anomalies [1,2,3]. FA is characterized as a rare genetic disorder of genomic instability that affects DNA repair and cell cycle regulation [4,5]. This instability results in inherited bone marrow failure syndrome (IBMFS), a condition that confers a high cancer risk, including, Myelodysplastic Syndrome, leukemia, typically acute myeloid leukemia (AML), and squamous cell carcinoma (SCC) [6,7,8].

Patients with FA present with well-defined and complex congenital abnormalities such as VACTERL-H (acronym for vertebral abnormalities, anal atresia, cardiac defects, tracheoesophageal, fistula, esophageal atresia, renal and radial abnormalities, limb abnormalities, and hydrocephalus), as well as PHENOS (acronym of skin pigmentation, café au lait spots, small head, small eyes, nervous system anomalies, otology, and short stature), Although not all patients present malformations or pancytopenia at birth, one-third of FA patients can be adults with normal physical appearances and normal hematopoiesis. In these patients, diagnosis occurs when they develop typical FA-associated cancers or through family studies [4]. 

FA is a rare disease with an incidence of 1 in 300,000 live births and a prevalence of 1–9 per million people. However, this frequency can vary according to the population, where the founder effect can be present [5]. FA is caused by the inactivation of at least one of 23 genes that play important roles in repairing DNA lesions, particularly interstrand crosslinks (ICLs). Mutations in 21 FA genes have been described and are shown to have an autosomal recessive effect [9,10]. The exceptions are mutations in the *FANCB* gene, which is known to be X-linked, and mutations in the FANCR/RAD51, which is an autosomal dominant mutation [11,12,13]. Regardless of their pattern of inheritance, the common feature generated by these mutations is chromosomal instability. 

Cytogenetic analysis using chromosomal breakage assays is the gold standard diagnostic assay for FA, using chromosomal breakage assays. The follow-up of the patients is performed by both classical and molecular cytogenetics. The diagnosis and follow-up of FA patients can be very challenging due to the biology and the corresponding impact on patient management. In this review, we discuss the current and emerging cytogenetic methods for the diagnosis, stratification in prognostic groups, and follow-up of FA patients, its impacts on patient care, and future perspectives on the use of cytogenetics and biomarkers of genomic instability for targeted therapies and clinical care.

## 2. Chromosome Breaking Assay—DEB, MMC, and Others

### 2.1. The Clinical Diagnosis Challenge

FA is a complex disease that presents with phenotypic heterogeneity, variable expressivity, and cannot be diagnosed based only on clinical presentation. In addition, its clinical manifestations are common to other diseases, such as Diamond–Blackfan Anemia (DBA), dyskeratosis congenita (DC), and Shwachman–Diamond syndrome (SDS), making the differential clinical diagnosis a challenge. Therefore, not all patients show clear clinical presentation [5], and only two-thirds of the patients harbor birth abnormalities [14,15,16]. The complexity of the clinical presentation of FA may lead to a delay in the final diagnosis, which directly impacts the patients’ outcomes. The correct and early diagnosis of the patients, by the gold standard diagnostic method (chromosome breakage assay), allows the parents to receive genetic counseling and make an informed and more secure family planning, considering the probability of having another child affected. Furthermore, the early diagnosis provides information regarding the need for therapy for developmental disabilities [16].

### 2.2. The History of Chromosome Breakage Assays and the Challenge in the Laboratory

The recognition of FA as a chromosomal instability disorder was made by the pioneer observations of Schroeder in 1964, where he noticed that lymphocytes of patients harbored significantly higher numbers of chromosome breakage in culture than their healthy counterparts. Therefore, Schroeder suggested the usefulness of this spontaneous phenomenon as a biomarker of FA [17,18]. However, later studies have shown that the presence of chromosome breakage could not be extensively applied as single cytogenetic evidence of the disease [19]. Later, a remarkable achievement was made by Schuler et al. (1969), demonstrating the possibility of adding DNA cross-linking agents in vitro, such as tetrametansulfonil-d-mannit (mannitol), to determine the increased breakage rate in FA samples [20]. Since then, other chemical substances other than mannitol have been proven to induce chromosome breakage such as cyclophosphamide, ionizing radiation, and nitrogen mustard. 

In the 1980s, Auerbach reported that hypersensitivity to the clastogenic effect of diepoxibutane (DEB) is a valuable discriminator for FA, suggesting a basic protocol for diagnosis of FA based on the addition of DEB in the peripheral blood of the patients [21]. In the same decade, Cervenka et al. developed the method based on the effect of the bifunctional alkylating agent mitomycin C (MMC) [22]. However, some authors still prefer to use DEB, considering its higher sensitivity, stability and specificity [6,16,21]. This was the agent used in our laboratory to diagnose 550 patients with FA (data not yet available). However, DEB is a carcinogen substance and a hygroscopic molecule that, upon contact with water, slowly loses activity. DEBs present a half-life of approximately four days due to its hydrolysis into 1,2,3,4-tetrahydroxybutane, a compound with no cross-linking activity. Likewise, MMC also has its limitations, requiring metabolic activation to become active as a cross-linking agent [19]. A detailed protocol with the specific concentration and index should be followed regardless of the choice of the agent to be utilized. In addition, the assay should be performed by experienced professionals with this diagnostic method [16,19,23]. 

One of the biggest challenges in chromosome breaking assay (CBA) is mosaicism [19]. Somatic mosaicism in FA (hereafter referred to as mosaicism) arises from reversion or other compensatory mutations in the hematopoietic stem cells/progenitor cells (HSPCs), from which a population of bone marrow and blood cells with a functional DNA repair capacity can arise. Patients’ cells with a reversion in a pathogenic FA mutation frequently present with two distinct blood cell populations: one sensitive to DNA-damaging agents and consistent with an FA diagnosis, and another resistant to these DNA-damaging (or clastogenic) agents [18,23,24,25].

According to a single comparative study, MMC appeared slightly more suitable for the assessment of lymphocyte mosaicism [26]. Other protocols have been developed to define mosaicism with both DEB and MMC alkylating agents [6,19,23]. Mosaicism can also be defined by cultivating another type of cells, such as fibroblasts [16]. If the CBA agrees with the anemia profile of AF, the next step is genetic testing to identify the specific variants causing AF. Genetic testing allows for accurate diagnosis and improves clinical care for patients and relatives who are heterozygous carriers of variants of the FA gene that confer an increased risk of malignancy. Currently, the test used for this analysis is the next-generation sequencing (NGS), which allows the detailed analysis of several genes simultaneously [9]. The early diagnosis of FA offers great advantages. 

The unrepaired breaks generally lead to cell cycle arrest but also increase the risk of clonal chromosomal abnormalities [19]. CBA can be challenging, considering that the phenotype of chromosomal instability can vary depending on several variables such as the type of the DNA cross-linking agent, small changes in concentration of the clastogenic agent, cell type (mosaicism), founder effect, and even the overlap of chromosome instability (CIN) levels with other diseases. 

## 3. The Search for Clonal Chromosomal Abnormalities

### 3.1. Classical Cytogenetics

Cytogenetics is the study of chromosome number and structure, chromosome banding permit single-cell and genome-wide analysis of chromosomal alterations, generating a foundational knowledge base of chromosome abnormalities and their clinical associations [27]. Genomic instability, the classical feature of FA, can increase the presence of clonal chromosome abnormalities, i.e., non-random and recurrent abnormalities. The first reports of clonal chromosomal abnormalities in the bone marrow of FA are from the end of the 1970s and 1980s. Generally, those abnormalities were associated with a preleukemic or during a leukemic clinical presentation [28] and included alterations on the short and long arm of chromosome 1 and 3, respectively, and monosomy of chromosome 7. In the 1990s, based on multicentric studies, the association of chromosomal abnormalities in the bone marrow to progressive bone marrow (BM) failure in FA patients was described [29,30,31,32,33,34]. Auerbach et al. (1991), in a comprehensive review of cytogenetic studies reporting FA associated with leukemia, reported that there was a high incidence of monosomy of chromosome (−7) and abnormalities in the long arm of chromosome 1 (ex. duplication of 1q). The most common chromosomal abnormalities found in de novo AML such as t(8;21), t(15;17), and inv(16) were not observed in FA patients with AML [28,34]. However, the role of chromosomal abnormalities found in FA patients without MDS or leukemia and/or the presence of transient chromosome clones was unclear [29,30,35]. Butturini et al. (1994), based on the analysis of 388 FA cases from the literature, reported that the risk of an FA patient to develop MDS or AML at 40 years of age was 52%. Additionally, the likelihood of presenting clonal chromosomal abnormalities at the age of 30 was 67%. Most of the cases reported presented with clonal chromosomal abnormalities concomitant with the disease progression to MDS or AML [32]. 

Alter (1996), in a comprehensive review of the literature on 1000 FA cases, reported that there was a progressive increase in risk of developing cancer as the patients aged. In the case of BM, it was suggested that an annual cytogenetic examination should be performed, in order to obtain a better understanding of the frequencies of clonal abnormalities and its prognostic value during BM failure [33]. In 2000, the same group, in a retrospective study of 41 FA cases showed an estimated 5-year survival of 0.4 with clonal abnormalities compared to 0.94 without clonal abnormalities [35]. In addition, it was suggested that the observation of floating or non-recurrent clones may be due to the small number of metaphases/cells analyzed; a more frequent classical cytogenetics evaluation of BM and Fluorescence in situ hybridization (FISH) would improve this analysis [36].

### 3.2. FA in the Age of Molecular Cytogenetics

A crucial step towards the development of FA cytogenetics was the application of molecular techniques, such as FISH. In this technique, a higher number of cells can be analyzed, considering that the presence of metaphases is not required, with the possibility of detecting clonal chromosome abnormalities in both metaphase and interphase nuclei. However, despite this advantage, FISH can only be used to detect known abnormalities [37], considering the need of well-defined FISH probes. Thruston et al. (1999) performed FISH in an analysis of a retrospective FA case of AML with monosomy of chromosome 7 and observed the presence of this abnormality 18 months before classical cytogenetics. If monosomy 7 is evidence of bone marrow transformation, early detection of a monosomy 7 clone by FISH would allow for early interventions, such as bone marrow transplantation, to help prevent or delay the onset of leukemia in these patients [38].

Remarkable achievements have been made in the study of FA with the development of Comparative Genomic Hybridization (CGH), a technique that allows the detection of chromosomal copy number alterations (CNA). The CGH technique, like FISH, does not require cell culturing, which is an advantage in FA, due to its decreased mitotic index [39]. The only need for genetic material for CGH is the patients’ DNA. Furthermore, CGH offers a global overview of the whole genome, providing data on gains and losses of DNA on the chromosome regions (CGH-chromosomic or conventional) or genomic regions (array-CGH). 

Tonnies et al. (2003) performed a retrospective serial analysis of 53 FA patients combining G-banding (Giemsa Chromosome Banding Technique, the most used chromosomal banding technique) and CGH, and demonstrated that 18 patients (72% of those with chromosomal abnormalities) had partial trisomy or tetrasomy of the long arm of chromosome 3 (also confirmed by FISH)—a number much higher than those already reported in the literature [40]. The low number of cases reported with 3q alterations can be due to the fact that the gain of this genetic material can be resultant of subtle chromosome translocations and/or small supernumerary marker chromosomes that cannot be resolved by its G-banding pattern analysis, thus were missed in the karyotyping [41]. Studies of transformed FA-AML strains demonstrated 3q26 gain as the only common gain, which resulted in the overexpression of the *EVT1* gene located in this chromosome region, as detected by CGH array and FISH*EVI1*. The overexpression of this gene in non-FA AML is associated with a low response to chemotherapy and poor prognosis [42]. The same group suggested, based on their data, that patients with 3q aberrations have an adverse risk factor, with poorer overall survival compared to FA patients without such aberrations [40]. 

Serial and prospective studies [7,32,39,42] began to consolidate information on chromosomal alterations in FA. In patients with abnormal clones, 77.8% had 1q, 3q, and −7. These three were responsible for 56% of the total chromosomal abnormalities [41]. It has been suggested that 3q may precede the onset of monosomy 7 and have shown an extremely poor overall survival rate compared to patients who did not present with this alteration [43,44]. Abnormalities in 3q and 7/7q- were associated with an increased risk of developing MDS or AML, suggesting that patients with −7 should be referred to BMT in the same way as if an expanding 3q clone is observed [43,45]. In contrast, the abnormality in 1q is described in morphologically normal BM and is also found in all stages of the progression of the hematological disease in FA. It can be seen as the only abnormality, but it also occurs in the presence of gains in 3q and other chromosome alterations [45,46]. Abnormalities involving the *RUNX1* locus at 21q have been associated with advanced MDS [7,47]. The abnormalities that have higher impact on patients with FA are not balanced and differ from the rearrangements commonly observed in non-FA AML patients.

### 3.3. Cytogenetics, Hematologic Conditions, and Treatment Intervention

In recent decades, several cytogenetic, hematological, and morphological studies were conducted in BM samples of FA patients significantly contributing to the clinical management of the patients. For example, the most optimum timing of monitoring BM was established, according to the patients age, which is of relevance considering that repeated BM aspirations are poorly tolerated in children, teenagers, and young adults [9]. The consensus is that a 1-year BM aspirate baseline is reasonable and should be adapted in response to changes in blood cell counts, signs of MDS, increased blast proportions, and/or cytogenetic evidence of clonal evolution. On the other hand, BM monitoring is likely to be slightly delayed in children younger than 10 years (except in *BRCA2/FANCD1* patients), given the rarity of the disease at this age and the relatively slow pace of clonal progression [47]. 

As far as treatment decisions, if the patient with FA already has a compromised hematological condition compatible with MDS or AML, therapeutic interventions should be performed promptly, such as bone marrow transplantation [45,46,47,48]. However, if the BM presents with a blast count of less than 5% in the marrow and less than 2% in the blood, cytogenetic monitoring may be indicative of the therapeutic proposals. Patients with normal cytogenetics and cytopenia should be followed up annually. If other isolated cytogenetic aberrations appear, such as 1q+, 20q-, 7p-, +8, 5q-, and 6p-, it is suggested that there should be close monitoring; however, if changes are observed in 3q+, −7/7q-, or complex karyotypes there is evidence of MDS [48]. The follow-up carried out in 246 patients at our institution corroborates the data in the literature that associate cytogenetic alterations in 3q, 7q/′7, and complex karyotype with MDS and AML. 

The impact of cytogenetic changes was analyzed by Ayas et al. (2013) in 113 patients with FA who underwent allogenic BMT. This study reported that patients who presented with only cytogenetic abnormalities had a better survival rate than patients with MDS and leukemia. These authors suggested that the ideal clinical approach is to perform BMT while the patient is in the aplastic phase, before developing any cytogenetic alteration, MDS or leukemia [49]. Wang et al. (2018), after monitoring the post-BMT of 73 patients, reported poor overall survival (OS) after 1 year, OS + 0% in patients who presented alterations in 3q or complex karyotypes vs. 45% when compared to those with no abnormalities [50]. 

**Figure 1:**
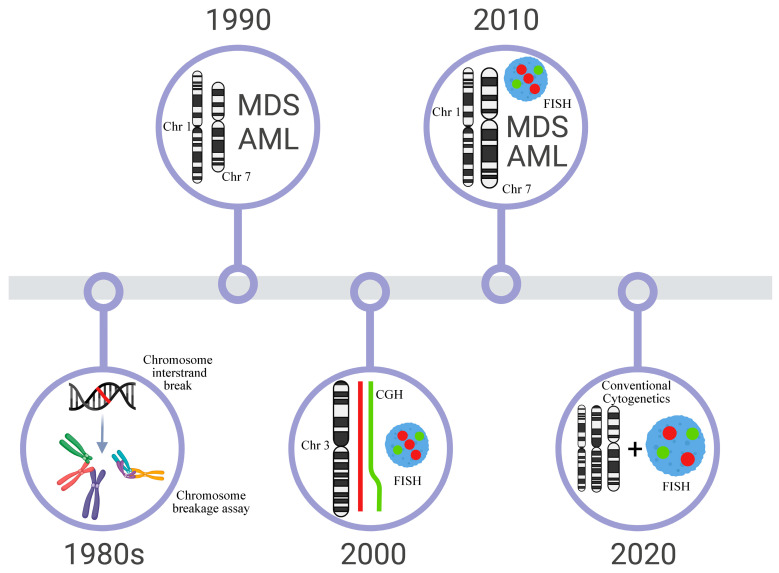
Timeline of the most important dates of Cytogenetics in FA. The first protocols for the diagnosis of FA with clastogenic agents were developed in the 1980s. In the 1990s, literature reviews and multicentric studies showed that changes in 1q+ and −7 were recurrent in the bone marrow and could be related to FA progression to MDS/AML. However, aberrations commonly observed in AML, t(8;21), t(15;17), and inv(16) were not seen in these patients. Molecular techniques (FISH, CGH, CGHarray, SNParray) were widely used in the 2000s and one of the big discoveries was the definition of extra material in 3q, a subtle alteration that is cryptically embedded in the karyotype. The next decade (2010) is marked by the reports of several follow-ups of patients with FA, which begin to outline the correlation of cytogenetic alterations with the evolution of the clinical and hematological condition. Since 2020, it has been observed that classical cytogenetics and FISH are the most commonly used techniques in patient follow-ups and can define highly relevant chromosomal alterations that guide therapeutic intervention for patients.

Altogether, the cytogenetic studies, indicate that non-balanced chromosome abnormalities, such as 1q gain, 3q gain, 7 monosomy, or 7q deletion, are recurrent in FA patients and present prognostic value as well as can indicate therapeutic intervention [49,50]. The involvement of these regions direct further molecular studies with higher resolution to define the involvement of genes in the development of BM failure and progression to MDS and AML (The most important achievements in the cytogenetics of FA are summarized in Figure 1). 

## 4. Other Cytogenetic Biomarkers in FA

Although DEB, G-banding, FISH, and CGH are the most known/used techniques to verify either chromosomal abnormalities or chromosomal instability, there are other cytogenetic biomarkers to measure/verify the later. Some of them are prognostic biomarkers in MDS and/or AML. FA is a heterogeneous disease with the shared feature of an abnormal FANC/BRCA pathway. Various biomarkers of genomic instability were found in FA cells from several complementation groups, examples of those biomarkers are Micronuclei, nuclear bridges, centrosome, and telomere dysfunction. Because of genomic instability, many cellular and molecular manifestations of this mechanism can occur, offering explanations for how the disease occurs, which can not only improve diagnosis and prognosis but also indicate druggable targets for the development of novel therapies.

### 4.1. Micronuclei

The micronuclei assay is one of the most-used techniques to detect genomic instability, and currently is mostly applied to genotoxicity evaluation. Fenech (2007) defined the micronuclei assay as the cytokinesis-block micronucleus cytome assay (CBMN). In this assay, micronuclei (MN), nuclei buds, and nuclear bridges, are quantified and indicative of genomic instability [51]. Furthermore, the mitotic index can be calculated by quantifying the number of binucleated cells [51]. Although it requires mitosis as conventional testing, the culture requires 24 h of mitotic arrest (inhibition of cytokinesis)—rather than only 20 min when using colchicine/colcemid for CBAs—therefore a larger number of cells are also analyzed (2000). 

German and Pugliatti-Crippa (1966) were the first to report the occurrence of MN in cells of FA patients and Bloom Syndrome (BSyn) in the absence of toxic treatments [52]. Since then, several research groups found increased levels of MN and nuclear bridges in several types of cells from these patients [53,54,55]. Maluf et al., 2001 reported that FA, when compared with HD, exhibited a higher frequency of MN and dicentric bridges, and suggested that the increased dicentric bridges in the same order as MN were the result of clastogenic events [53]. Interestingly, Naim et al. (2009) demonstrated that the FANC pathway has a role in preventing the formation of MN and chromosomal abnormalities. They demonstrated that the FANC pathway loss-of-function causes chromosomal abnormalities, lagging chromosomes, MN, and anaphase bridges (the two later are detected in CBMN), suggesting that this pathway is not only involved in DNA repair but also in chromosome segregation, differing FA from other instability diseases such as BSyn [54]. *FANCD2* localizes in the central connection points of these bridges [56]. These bridges were suggested as a cause of cytokinesis failure leading to binucleated cells [56]. The cells with two nuclei may lead to BM failure in FA. In the study of Nalepa et al. 2013, high levels of MNs were found in the cells from patients from several FA complementation groups [55]. Together, these studies suggest MN as an important cytogenetic feature of FA cells. 

Based on the previous studies of the occurrence of MN in FA [53,54,55,56], Francies et al. (2018) proposed an MMC protocol combined with MN as an alternative technique for the diagnosis of FA patients. In this study, these authors used MMC to induce MN formation and observed significant differences in MN among FA homozygotes, FA heterozygotes, and controls. They also tested the effect of ionizing radiation on MN formation in FA patients, their parents, and control individuals. FA patients exhibit higher levels of radiosensitivity in the MN assay when compared to parents and controls. Although this assay cannot be suggested as a biomarker for diagnosing FA, the MN would be important in proving information on radiosensitivity before being referred to ionizing radiation treatment for cancer [57]. 

MN is also an important biomarker in the most common neoplasms found in FA patients such as MDS and AML. MN has been suggested as one of the hallmarks of CIN with prognostic value in these hematological malignancies. Wang et al. 2013 found that levels of MN formation in blood lymphocytes of AML patients provide prognostic value on disease progression [58]. Huh et al. (2016) linked gene amplification to MN formation in leukemic blasts of MDS and AML. The *MYC* and *MLL* gene amplifications were present in the form of MN in these cells [59].

MN analysis presents advantages when compared to conventional CBA. Diagnosing FA by CBA (DEB or MMC) in metaphases can be laborious and require professionals with extensive experience. Furthermore, MN analyses can be automated by several techniques such as flow cytometry and automated fluorescence microscopy. It seems to be a reliable technique but requires further studies to evaluate the sensitivity and specificity of this test in FA patients and parents with other genotypes/mutated FANC genes [57]. There is the need to compare how mosaicism can impact MN levels. It also can be combined with FISH [51], which may provide explanation for specific chromosomal abnormalities found in FA. Furthermore, the data from Francies et al. were obtained in a specific cohort, which could lead to an influence of the founder effect. MN incidence should also be compared among the genomic instability diseases that are challenging to discriminate from FA. How MN levels change in FA cells during the course of MDS or AML manifestation remains elusive.

### 4.2. Centrosome Dysfunction

Centrosomes are key structures for the proper segregation of chromosomes during cell division. These cellular structures are known as microtubule-organizing centers [60]. Centrosome dysfunction are one the of most common biomarkers of genomic instability and cancer [61,62]. This feature is key mechanism leading to aneuploidy in several cancer types [63,64,65,66]. Centrosome dysfunction can be numerical, such as centrosome amplification, presenting dysregulation in the duplication cycle, overduplication, mitotic chaos, and entosis. There are also structural centrosome abnormalities caused by changes in the amount of centrosome constituents, irregular localization of core proteins, and abnormal binding among core proteins [62,67,68].

Several studies have demonstrated that nearly all FA complementation groups are associated with centrosome dysfunction [55,69,70,71,72]. Nalepa et al. (2013) found that eight of the FA proteins (FANCA, FANCB, FANCD1, FANCD2, FANCE, FANCG, FANCL, and FANCN) particularly localize to centrosomes during mitosis. However, the localization of FANCC and FANCA to the mitotic spindle depends on cell cycle. Primary FA fibroblasts exhibited higher number of centrosomes when compared to controls [55]. FANCD1 (also known as BRCA2), another FA protein, is a key element in the preservation of centrosomes and colocalizes with these structures. Loss of FANCD1/BRCA2 leads to both MN formation and centrosome amplification [69,70,71]. FANCA also localizes to centrosomes and participates in the integrity of these structures [72]. In addition, it was demonstrated that FANCA is crucial in the regulation of centrosomes-associated spindle assembly [55,73].

Centrosomes are a key component of the DNA damage response, as shown when cells are exposed to MMC or cis-platin (DNA interstrand crossing linking agents) they exhibit centrosome amplification. The FancJ protein regulates normal centrosomes cycle and centrosome amplification caused by ICL [74,75]. Furthermore, loss of function of *FancJ* occurs in FA and breast cancer indicating that such abnormality is involved in centrosome dysfunction as a cancer suppressor feature [76,77].

Centrosome dysfunction was reported in both MDS and AML. Centrosome abnormalities are higher in MDS patients with cytogenetic changes and predict transformations to AML [78,79]. Interestingly, based on prognostic information provided by karyotype changes, centrosome abnormalities were correlated to cytogenetically defined risk groups, suggesting their role as prognostic biomarkers [80]. This observation suggests a possible role of this mechanism, not only in the induction of chromosomal abnormalities but also in clonal evolution. Therefore, centrosome dysfunction in MDS-FA and AML-FA, if investigated in more detail, could provide prognostic information. Key proteins involved in centrosome maintenance were found to be abnormal in both MDS and AML [81]. In addition, these protein abnormalities were also correlated to an increased number of chromosomal abnormalities. 

Several of the most important proteins affected in FA are associated with centrosome maintenance and the most common mutations in this disease lead to centrosome dysfunction. The abnormal FANCD1/BRCA2 is not only involved in centrosome amplification but also in the occurrence of MN, suggesting an overlap of biomarkers of genomic instability in certain complementation groups. Centrosome dysfunction can be both a cause and consequence of genomic instability, however further studies are needed to better clarify the role of these phenomenon in FA cells. The studies showing the role of the centrosome in MDS and AML could be applied in FA patients who evolved to these malignancies. Centrosome dysfunction and AML were associated with the increased occurrence of chromosomal abnormalities. Although most of these observations were obtained from studies in older patients because of the association between age and genomic instability, the BM failure was also an important variable in these patients. Since BM failure is one of the main features of FA, research efforts to understand the role of centrosome dysfunction in the emergence of these malignancies in FA patients seems to be promising. If better understood and validated as a prognostic biomarker, its application is not so dependent on technical training such as other cytogenetic techniques. Therefore, a better understanding of how this genomic instability occurs in FA can be very promising. 

### 4.3. Telomere Dysfunction

Telomere dysfunction is shared by many genomic instability diseases, leading to abnormal levels of apoptosis and increasing the risk of cancer [82,83,84,85]. FA peripheral blood lymphocytes generally present shorter telomeres, telomere loss/breaks, and high levels of telomeric association [86,87,88]. Several causes of telomere dysfunction in FA have been proposed, such as spontaneous telomere sequence breaks, shortening resulting from replication, accumulative breaks derived from abnormal DNA repair, and compromised responses to oxidative stress [89,90,91,92,93,94,95].

Several studies pointed out that FA proteins participate in the maintenance of telomere length [96,97]. Fan et al. (2009) demonstrated that in ALT (alternative lengthening of telomeres) cells, the colocalization of FANCD2 to the inherent telomeric protein TRF1—one of the proteins in the shelterin protein complex of telomeres—relied on FANCA and FANCL. It was also observed that FANCA and FACD2 depletion also leads to telomere loss and decreased telomere sister chromatid exchange. This suggests the key role of FACD2 in telomere maintenance [96]. Interestingly, even when exposed to MMC, hematological and non-hematological cells of FANCG-deficient mice do not show evidence of telomere dysfunction [98]. Insufficiency of FANCC also does not directly cause telomere dysfunction, however it plays a role in telomere attrition and the lack of telomerase and short telomeres increases telomere sister chromatid exchange. This suggests that FANCC deficiency leads to an accelerated telomere shorting during high levels of hematopoietic cells replacement [99]. Telomere maintenance is essential in BM cells in a BM failure disease such as FA. Furthermore, SLX4 is frequently mutated in FA. The SLX4 protein plays diverse roles in genomic stability, one of which is maintaining telomere length [97]. 

Telomere dysfunction is also present in DC, a disease with overlapping genomic instability features with FA. When compared to DC, FA telomeres are not extremely short [100]. The main mutations of FA are not directly involved in telomere biology [101]. In DC, telomere dysfunction is a direct result of the mutated genes; in FA it seems to be more likely the result of hypersensitivity to oxygen leading to increased oxidative damage at telomeric structures [95]. DC share with FA the increased risk of developing MDS and AML. Telomere dysfunction was extensively studied in these myeloid malignancies and this genomic instability hallmark was associated with chromosomal abnormalities, disease progression, and therapy resistance [102,103,104].

A more precise description of how telomere dysfunction occurs in this disease is a key step for the future use of this feature as a prognostic biomarker in FA. The recognition of this genomic instability feature/mechanism as a cancer hallmark also suggests the importance of a better comprehension of this topic. This dysfunction can also lead to extreme levels of apoptosis which, in turn, can lead to BM failure, one of the main characteristics of FA. Abnormal mechanisms of telomere maintenance of hematopoietic cells may be crucial in the development of BM failure in FA. Differentiating how FA proteins act in telomere biology has only started. Additional experimental evidence in their role is needed to determine the use of telomere dysfunction as a biomarker in FA. It is promising, since telomere dysfunction has a role in cancer and aging, which has led numerous research groups to develop automated approaches for verifying and quantifying this genomic instability feature.

## 5. Conclusions

FA is a disease of chromosomal/genomic instability. Cytogenetics comprise one of the key steps in adequately diagnosing this disease. The rationale is based on the increased risk of malignancy because of CIN. However, the diagnosis can be very challenging. Some of the main features of FA are shared with other genomic instability diseases. Discriminating is crucial and not always easy. Although DEB/MMC is considered the “gold” standard in FA, the cytogenetic diagnosis also includes chromosome banding and molecular cytogenetics. Each technique has its advantages, limitations and is indicated to only a specific part of the clinical practice.

**Figure 2:**
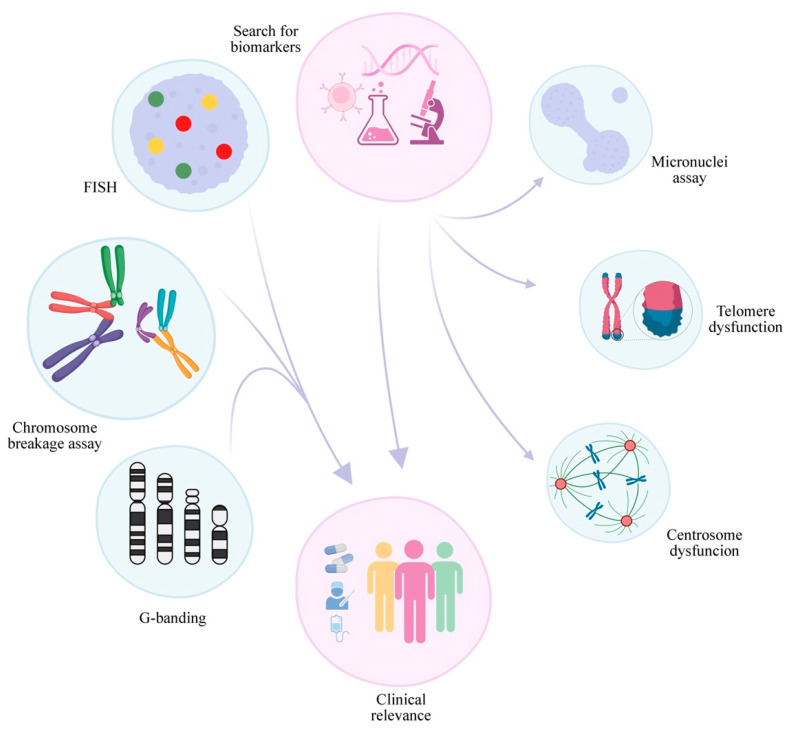
Cytogenetics in FA: summary of conclusions and future perspectives. The progress of cytogenetics in FA in recent decades has led to the establishment of CBA breakage as the “gold” standard which, combined with classical and molecular cytogenetics, provides valuable information for patient management and treatment. The search for new biomarkers in FA and a better comprehension of those already identified, including abnormalities in binucleated cells generated in the micronuclei cytome assay and telomere dysfunction, may lead to a better understanding of the disease and provide better treatments in the future.

Moreover, a crucial part of FA research progress is how to follow up with the patients. Although there are established guidelines, there are still many open questions. Thus, the study of the already mentioned potential biomarkers and the search for more new ones must continue. As new biomarkers are discovered, more clinical approaches can be tested and provide the patient with a more personalized approach (Figure 2). Several of the biomarkers of genomic/chromosomal instability present in FA anemia cells are similar in MDS and AML, harboring prognostic value. However, significant effort must be employed to clarify how these biomarkers occur in the different complementation groups and how they change during the emergence of cancer in FA patients. 

## 6. Future Perspectives

The detailed comprehension of how cytogenetic/genomic instability occurs in FA is a challenge. As described above, each complementation group is involved in different processes in the maintenance of genomic instability. Although many questions remain, the accumulated data on chromosomal/genomic instability biomarkers in nearly all FA groups suggest an important field to be explored. Most of the biomarkers mentioned are not laborious and can even be automated. These biomarkers are involved in many aspects of cancer, aging, and other phenomena that have captured the attention of the research community; therefore, many approaches for application techniques for these biomarkers are available. 

Efforts to understand how chromosomal/genomic instability occurs in other types of FA cells are needed. Most of what is known has been limited to blood cells and fibroblasts. Alternative cell types for the diagnosis have been proposed. Chromosomal/genomic instability can be both a tumor suppressor and a tumor promoter. This ambivalent characteristic has been exploited in many cancer models. Some of the genomic/chromosomal instability features of FA could be exploited clinically by targeting the mechanisms and by products of the instability. We still do not know exactly how the genes present in the most common FA chromosomal abnormities affect predisposition to MDS and AML. It remains unclear how FA cases that evolve to MDS and AML differ from other subtypes (AML de novo) of these malignancies. Another crucial step for using the mentioned biomarkers is more accurate discriminations on the biomarkers’ occurrence levels shared with other genomic instability diseases.

## Data Availability

Not applicable.
